# *PLAA* Mutations Cause a Lethal Infantile Epileptic Encephalopathy by Disrupting Ubiquitin-Mediated Endolysosomal Degradation of Synaptic Proteins

**DOI:** 10.1016/j.ajhg.2017.03.008

**Published:** 2017-04-13

**Authors:** Emma A. Hall, Michael S. Nahorski, Lyndsay M. Murray, Ranad Shaheen, Emma Perkins, Kosala N. Dissanayake, Yosua Kristaryanto, Ross A. Jones, Julie Vogt, Manon Rivagorda, Mark T. Handley, Girish R. Mali, Tooba Quidwai, Dinesh C. Soares, Margaret A. Keighren, Lisa McKie, Richard L. Mort, Noor Gammoh, Amaya Garcia-Munoz, Tracey Davey, Matthieu Vermeren, Diana Walsh, Peter Budd, Irene A. Aligianis, Eissa Faqeih, Alan J. Quigley, Ian J. Jackson, Yogesh Kulathu, Mandy Jackson, Richard R. Ribchester, Alex von Kriegsheim, Fowzan S. Alkuraya, C. Geoffrey Woods, Eamonn R. Maher, Pleasantine Mill

**Affiliations:** 1 MRC Human Genetics Unit, Institute of Genetics and Molecular Medicine, University of Edinburgh, Edinburgh EH4 2XU, UK; 2 Cambridge Institute for Medical Research, University of Cambridge, Cambridge CB2 OXY, UK; 3 Department of Medical Genetics, University of Cambridge, and Cambridge NIHR Biomedical Research Centre, Cambridge Biomedical Campus, Cambridge CB2 OXY, UK; 4 Centre for Integrative Physiology, University of Edinburgh, Edinburgh EH8 9XD, UK; 5 Euan McDonald Centre for Motor Neuron Disease Research, University of Edinburgh, Edinburgh EH16 4SB, UK; 6 Department of Genetics, King Faisal Specialist Hospital and Research Center, Riyadh 11211, Saudi Arabia; 7 MRC Protein Phosphorylation and Ubiquitylation Unit, University of Dundee, Dundee DD1 5EH, UK; 8 West Midlands Regional Genetics Service, Clinical Genetics Unit, Birmingham Women’s Hospital, Birmingham B15 2TG, UK; 9 Centre for Genomic and Experimental Medicine, Institute of Genetics and Molecular Medicine, University of Edinburgh, Edinburgh EH4 2XU, UK; 10 Edinburgh Cancer Research UK Centre, Institute of Genetics and Molecular Medicine, University of Edinburgh, Edinburgh EH4 2XU, UK; 11 Systems Biology Ireland, University College Dublin, Dublin, Ireland; 12 Electron Microscopy Research Services, Newcastle University, Newcastle NE2 4HH, UK; 13 Patrick Wild Centre, University of Edinburgh, Edinburgh EH8 9XD, UK; 14 Department of Pediatric Subspecialties, Children’s Hospital, King Fahad Medical City, Riyadh 11211, Saudi Arabia; 15 NHS Lothian, Department of Paediatric Radiology, Royal Hospital for Sick Children, Edinburgh EH9 1LF, UK; 16 Centre for Cognitive and Neural Systems, University of Edinburgh, Edinburgh EH8 9JZ, UK; 17 Department of Anatomy and Cell Biology, College of Medicine, Alfaisal University, Riyadh 11533, Saudi Arabia; 18 Saudi Human Genome Program, King Abdulaziz City for Science and Technology, Riyadh 11442, Saudi Arabia

**Keywords:** ubiquitin, endolysosomal trafficking, autophagy, synaptic vesicle recycling, synapse, microcephaly, cerebellum, Phospholipase A2-activating protein, Ufd3, seizures

## Abstract

During neurotransmission, synaptic vesicles undergo multiple rounds of exo-endocytosis, involving recycling and/or degradation of synaptic proteins. While ubiquitin signaling at synapses is essential for neural function, it has been assumed that synaptic proteostasis requires the ubiquitin-proteasome system (UPS). We demonstrate here that turnover of synaptic membrane proteins via the endolysosomal pathway is essential for synaptic function. In both human and mouse, hypomorphic mutations in the ubiquitin adaptor protein *PLAA* cause an infantile-lethal neurodysfunction syndrome with seizures. Resulting from perturbed endolysosomal degradation, *Plaa* mutant neurons accumulate K63-polyubiquitylated proteins and synaptic membrane proteins, disrupting synaptic vesicle recycling and neurotransmission. Through characterization of this neurological intracellular trafficking disorder, we establish the importance of ubiquitin-mediated endolysosomal trafficking at the synapse.

## Introduction

The ubiquitin code involves post-translational modification of target proteins by covalently attaching ubiquitin (Ub) via lysine residues, coordinating diverse and essential processes. Disruption of Ub-mediated signaling causes a range of disease phenotypes including cancer, immune deficiency, diabetes, and neurodegeneration.^2^ The ubiquitin code is complex, with polyUb chains formed by covalent attachment to lysines (K) present on ubiquitin itself. The precise lysine residue used in linkages dictates the structure of the chains, altering the outcome for the target protein; Lys48 (K48) polyUb chains are primarily involved in proteasomal targeting, whereas Lys63 (K63) polyUb chains are involved in signaling, DNA repair, or endocytosis. Interpretation of this code is mediated by diverse Ub-binding proteins via their Ub-binding domains (UBDs), which show differential affinity to the various ubiquitin modifications.^3^

Presynaptic terminals undergo extensive membrane remodeling during synaptic activity, with repeated rounds of exo-endocytosis of synaptic vesicles (SVs). Sustained neurotransmission depends on high-fidelity sorting of synaptic proteins during SV recycling.^4^ This is essential for neural function because too little or too much of critical synaptic membrane proteins, including SV2 and SNAP25, results in seizures, synaptic dysfunction, and early lethality.^5^, ^6^, ^7^, ^8^, ^9^ How this process is regulated and the involvement of ubiquitin signaling remains unclear.

Ubiquitin signaling has long been known to play a role in synapse development and plasticity,^10^ but this is generally attributed to the dependence of synaptic proteostasis on the UPS.^11^, ^12^, ^13^ Indeed, acute depolarization of isolated synaptosomes causes a global decrease in Ub-modified proteins, highlighting the rapid turnover rate of polyUb-proteins presynaptically.^14^ In the case of neurodegenerative disease, proteotoxic accumulations of Ub are noted as a hallmark,^15^ although in several of these disorders, the earliest symptoms are synaptic dysfunction.^16^ Exactly how this ubiquitin signaling is acting locally at the synapse to regulate normal presynaptic function is unclear. An alternative to the exclusively UPS-based model of synaptic proteostasis was first suggested decades ago whereby synaptic membrane protein turnover could involve endolysosomes.^17^ However, subsequent work has focused on exploring neuronal-specific “sort-and-degrade” mechanisms for synaptic cargos via endosomal intermediates whereupon the tie to Ub signaling has been obscured.^18^, ^19^ Results from two recent cell-based studies suggest that Ub signaling in synaptic vesicle turnover needs revisiting.^1^, ^20^

Here we describe, in four families, a severe early-onset neurodysfunction syndrome characterized by profound developmental delay and seizures resulting from homozygous mutations in the gene encoding ubiquitin binding protein Phospholipase A_2_ Activating Protein (*PLAA* [MIM: 603873]). PLAA binds ubiquitin through two UBDs, a high-affinity WD40 β-propeller, and a low-affinity PFU (PLAA family of Ub-binding) domain.^21^, ^22^ PLAA is the highly conserved ortholog of yeast Ufd3/Doa1 (FungiDB: YKL213C; 40% protein ID), which has well-documented roles in targeting ubiquitylated proteins for degradation through the ubiquitin-proteasome system (UPS) via interactions with the segregase p97/CDC48,^21^, ^23^ as well as regulating levels of free ubiquitin.^24^ Together with CDC48, Doa1 is suggested to play roles in diverse degradative processes including mitochondria-associated degradation (MAD)^25^ and starvation-induced degradation of mature ribosomes (ribophagy).^26^ Independent of its role in regulating free ubiquitin, Doa1 was shown to be required for sorting specific ubiquitylated cargos to late endosomes/MVBs for lysosomal degradation.^27^, ^28^ Despite the proposed roles for yeast ortholog Ufd3/Doa1 in Ub-dependent trafficking, the role of mammalian PLAA remains unclear.

We demonstrate that PLAA is required for the Ub-mediated sorting of membrane proteins from the early to late endosome, targeting them for lysosomal degradation. Unlike in yeast, in the absence of PLAA we see no changes in free ubiquitin, although we observe the specific accumulation and altered processing of a subset of K63-ubiquitylated proteins. Importantly, we demonstrate that PLAA is essential for neural function, through dual roles of (1) regulating post-endocytic trafficking of signaling receptors necessary for neural development and (2) directing sorting of synaptic vesicle (SV) components during recycling, essential for synaptic function. This work demonstrates that ubiquitin-dependent endolysosomal proteostasis at the synapse is essential at the level of complex neural networks in humans and mice.

## Material and Methods

### Subject Ascertainment

Affected individuals in families A–C were ascertained following referral to the local NHS Regional Clinical Genetics Service. At the time of referral and clinical assessment, no clinical laboratory molecular genetic testing was available to confirm the working clinical diagnosis in each family and the families were recruited to a research study to investigate the molecular basis of the disorder. Research was conducted according to the principles expressed in the Declaration of Helsinki and was approved by the local Research Ethics Committees. All participants provided informed consent for the collection of samples and subsequent analysis. Family D was recruited with informed consent under an IRB-approved protocol (KFSHRC).

### Gene Mapping

Genome-wide genetic linkage studies were performed using Affymetrix SNP arrays (10k array in A-IV-6 and A-IV-7 and 250k in A-IV-1 and A-IV-8) in affected individuals (as described previously^29^ and Axiom in family D). Homozygous regions ≥2 Mb were further analyzed by typing microsatellite markers in all family members from whom DNA was available.

### Exome Sequencing

Exome sequencing was performed on one affected child from all families using the SureSelect Human All Exon 50Mb Kit (Agilent Technologies UK, Cat. No G3370A). Sequencing was performed with the SOLiD4 System (Applied Biosystems) with 50 bp fragment reads (in families B and C) or the Illumina Analyzer IIx with 76 bp paired end reads (in families A and D). Raw sequencing reads were mapped to the GRCh37 reference human genome and changes compared to this reference sequence identified. Analyses focused on non-synonymous coding, nonsense, splice site variants, and indels involving exons. Potentially pathogenic mutations were identified based upon being unknown variants or those where the rare allele frequency was <1%, how well the site was conserved throughout evolution, and re-examination of the sequence reads containing potential mutations using the Integrated Genome Viewer. Analysis of the 84 genes in the candidate interval in family A revealed only one rare potentially pathogenic variant in *PLAA* (Table S2). This c.68G>T mutation in *PLAA* was found to segregate correctly by Sanger sequencing in families A, B, and C.

### Generation of Mouse Models

Animals were maintained in SPF environment and studies carried out in accordance with the guidance issued by the Medical Research Council in “Responsibility in the Use of Animals in Medical Research” (July 1993) and licensed by the Home Office under the Animals (Scientific Procedures) Act 1986. *Plaa*-null mice (*Plaa*^*tm1(NCOM)Cmhd*^, MGI:4880046) were generated as detailed in Figure S2. *Plaa*^*G23V/+*^ mice (*Plaa*^*em1Pmi*^ MGI:5828117) were generated using the CRISPR-nickase Cas9 system as described in Figure S4. Genotyping was performed using primers detailed in Table S9.

### Mouse Phenotyping

#### Gait Analysis

*Plaa*^*G23V/G23V*^ and wild-type littermate control mice were videoed in a custom-made gait analysis chamber. Each paw placement in 2D was recorded manually using a custom macro written for ImageJ (code available upon request). Parameters such as step length, step time, and number of steps taken were calculated in Excel. Mice were analyzed at a variety of ages from 4 to 16 weeks, and altered gait was evident at all ages tested.

#### Grip Strength Test

Mice grip a metal grid attached to a sensor (Bioseb) with either their forelimbs only or both fore- and hindlimbs and maximal grip strength was recorded.

#### LacZ Staining

Adult *Plaa*^*+/−*^ brains were fixed briefly in 4% PFA/PBS for 1 hr at 4°C, then 200 μm vibratome sections were cut and collected into PBS. E11.5 (embryonic day) *Plaa*^*+/−*^ embryos were fixed in 4% PFA/PBS for 20 min at 4°C. LacZ staining was performed as described.^30^

#### Magnetic Resonance Imaging

Brains from 3-month-old animals were fixed in 4% PFA in the skull, with septum broken to aid penetration, for 5 days at 4°C, rotating. Brains were incubated in contrasting agent Dotarem (Guebert) for 7 days. Imaging was performed on a 7-Tesla small animal imaging system controlled by an Agilent VnmrJ 4 console (Agilent Technologies). The specimen was placed in the center of a 26-mm volume coil (Rapid Biomedical) used for radiofrequency transmission and reception. Structural imaging was accomplished using a 3D gradient echo sequence with repetition time = 30 ms, echo time = 6.22 ms, flip angle = 50°. The acquisition matrix was 512 × 192 × 192 over a 40 mm × 17 mm × 17 mm field of view, resulting in an image resolution of 78 × 88 × 88 μm. The images were analyzed in Fiji, using manual segmentation.

#### Transcriptomics

*Plaa*^*+/+*^ or *Plaa*^*G23V/G23V*^ brains (n = 3 per genotype) were subdissected into caudal (including cerebellum, medulla, and pons) and rostral (cerebrum) regions. Total RNA was extracted using QIAGEN RNAeasy Lipid mini kits, including on-column DNase digestion as per manufacturer’s direction. Expression analysis was performed using the Affymetrix GeneChip Mouse Transcriptome Array 1.0 (Aros) and analyzed using Affymetrix Transcriptome Analysis Software. Results were confirmed by qPCR using Roche Universal Probe Library (UPL) System on a LightCycler 480. Primer and probe sequences are available upon request.

#### Neuromuscular Junction Morphological Analysis

Protocol for NMJ morphological analysis is described in the legend of Figure S6. A minimum of 50 NMJs from a minimum of 3 fields of view were quantified per muscle. Pre-synaptic accumulations were defined as near-spherical neurofilament-positive structures that occurred at the pre-synaptic terminal. Sprouts were defined as a neurofilament-positive process that extended from the pre-synaptic terminal. Methods for NMJ transmission electron microscopy are described in the legend of Figure S6.

#### NMJ Electrophysiology

Levator auris longus (LAL) muscles were dissected into HEPES-buffered mammalian physiological saline (MPS; composition in mM: Na^+^ 158, K^+^ 5, Ca^2+^ 2, Mg^2+^ 1, Cl^−^ 169, glucose 11, HEPES 5 [pH 7.2–7.4]). Intracellular recordings of spontaneous miniature endplate potentials (MEPPs) and supramaximal nerve-evoked EPPs (<10V, <0.2 ms stimulation pulses) were recorded using glass microelectrodes filled with KCl (3M; resistance typically 20–30 MΩ) after selectively blocking muscle action potentials using μ-conotoxin GIIIB, as described previously.^31^, ^32^ Data were acquired and analyzed using a combination of WinWCP (Strathclyde Electrophysiological Software), Spike-2 (Cambridge Electronic Design), Minianalysis (Synaptosoft), pCLAMP (Molecular Devices), and Prism (Graphpad) software.

#### NMJ Synaptic Vesicle Recycling

Motor nerve terminals in isolated LAL muscles (dissected as above) were bathed in MPS containing FM1-43 (8 μM) together with rhodamine (TRITC)-α-bungarotoxin (5 μg/mL; both from ThermoFisher Scientific) to counterstain endplate acetylcholine receptors. One of the innervating intercostal nerves was stimulated continuously at 20 Hz for 10 min, and then NMJs were imaged, after washing for >30 min in MPS, using confocal microscopy as described previously.^33^, ^34^ Images were processed for overall brightness, contrast, and gamma only using Photoshop (Adobe) and then analyzed using ImageJ.

#### Acute Cerebellar Slices

Cerebella were dissected from 3-month-old mice into ice-cold modified artificial cerebrospinal fluid (ACSF) containing (in mM): 60 NaCl, 118 sucrose, 26 NaHCO_3_, 2.5 KCl, 11 glucose, 1.3 MgCl_2_, and 1 NaH_2_PO_4_ at pH 7.4 when bubbled with 95% O_2_:5% CO_2_. The cerebellar vermis was glued to the vibratome cutting platform (Leica Biosystems) with cyanoacrylate adhesive. 200 μm-thick sagittal slices were cut and incubated for 30 min at 30°C in standard ACSF composed of the following (in mM): 119 NaCl, 2.5 CaCl_2_, 26 NaHCO_3_, 2.5 KCl, 11 glucose, 1.3 MgCl_2_, and 1 NaH_2_PO_4_ at pH 7.4 when bubbled with 95% O_2_:5% CO_2_. Slices were stored at room temperature until required, then transferred to a submerged recording chamber and superfused with standard ACSF (3–5 mL min^−1^) at 30°C.

#### Purkinje Cell Electrophysiology

Whole-cell recordings were made from Purkinje cells voltage-clamped at −70 mV using thick-walled borosilicate glass pipettes pulled to 3–5 MΩ. For recording mIPSCs, the internal solution contained (in mM): 150 CsCl, 1.5 MgCl_2_, 10 HEPES, 0.1 cesium BAPTA, 2 sodium ATP, 0.4 sodium GTP, 5 QX-314 at pH 7.3. NBQX (10 μM) and tetrodoxin (300 nM) were added to the ACSF to isolate mIPSCs. Series resistances were <15 MΩ and were compensated for by 85%. Currents were filtered at 6 kHz and sampled at 10 kHz. Data was acquired and analyzed using pClamp 10 (Axon Instruments).

#### Purkinje Cell Single-Cell Imaging

Whole-cell recordings from Purkinje cells were performed with an internal solution containing (in mM): 0.2 Lucifer Yellow (Sigma, L0144), 0.02 Alexa FluorAR 568 hydrazide (Invitrogen, A-10441), 125 K-gluconate, 15 KCl, 10 HEPES, 5 EGTA, 2 MgCl_2_, 0.4 NaGTP, 2 NaATP, and 10 Na-phosphocreatine at pH 7.4. Purkinje cells were voltage-clamped at −60 mV for 25–30 min and complete cell filling was monitored by Lucifer Yellow fluorescence. Slices were then fixed with 4% paraformaldehyde in 0.1 M phosphate buffer (pH 7.4) overnight at 4°C. Slices were washed twice in 0.1 M phosphate buffer (pH 7.4) and twice in dH_2_O then stored in Vectashield (Vector Laboratories) at 4°C. Slices were wet-mounted with Vectashield onto 0.13 mm thick borosilicate glass and imaged using a Zeiss inverted LSM510 confocal microscope. Dendritic length and surface area was analyzed using ImageJ software (NIH).

#### Mass Spectrometry

*Plaa*^*+/+*^ or *Plaa*^*G23V/G23V*^ brains (n = 3 per genotype) were subdissected into caudal (including cerebellum, medulla, and pons) and rostral (cerebrum) regions and were homogenized in 100 mM Tris-HCl (pH 7.5) in presence of protease and phosphatase inhibitors (Roche). Samples were homogenized with an Ultra-Turrax T25 High-Speed Homogenizer System for 1 min on ice and SDS was added to a final concentration of 2% SDS. Lysates were sonicated and clarified by centrifugation. Samples were processed by a multi-protease FASP protocol as described.^35^ In brief, the SDS was removed and the proteins were first digested with Lys-C (Wako) and subsequently with Trypsin (Promega) with an enzyme to protein ratio (1:50). 10 μg of Lys-C and Trypsin digests were loaded separately and desalted on C18 Stage tip and eluates were analyzed by HPLC coupled to a Q-Exactive mass spectrometer as described previously.^36^ Peptides and proteins were identified and quantified with the MaxQuant software package, and label-free quantification was performed by MaxLFQ.^37^ The search included variable modifications for oxidation of methionine, protein N-terminal acetylation, and carbamidomethylation as fixed modification. Peptides with at least seven amino acids were considered for identification. The false discovery rate, determined by searching a reverse database, was set at 0.01 for both peptides and proteins. All bioinformatic analyses were performed with the Perseus software. Intensity values were log-normalized, 0-values were imputed by a normal distribution 1.8 π down of the mean and with a width of 0.2 π. Statistically significant variance between the sample groups was tested by a permutation-based FDR approach and a Student’s t test with a p value cut-off of 0.01. Total proteomic data are available via ProteomeXchange with identifier PXD003140 and are summarized in Table S6.

#### Synaptic Preparations

Cerebella were lysed in SYN-Per Synaptic Protein Extraction Reagent (ThermoScientific) according to manufacturer’s instructions.

### Homology Modeling and Mutation Analysis

The target WD40 seven-bladed β-propeller domain of human PLAA was modeled by homology based upon the high-resolution crystal structure template of yeast Doa1-WD40 (PDB: 3ODT; chain B, 1.35 Å resolution).^22^ The two sequences share 43% sequence identity and 60% similarity. The target-template alignment was generated based upon an initial multiple sequence alignment of related-divergent orthologs using PROMALS^38^ and manually edited to optimize positions of secondary structure elements and gaps. A total of 50 models were built for the human PLAA-WD40 using Modeler 9v12^39^ and the model with the lowest objective function score was selected. The selected 3D model was checked for valid stereochemistry using RAMPAGE^40^ (98% of residues in favored and allowed regions of the Ramachandran plot); the packing quality was evaluated using the WHATIF server^41^ (average quality control score −0.52; to place this in context, incorrect models give scores of < −3.0; lower quality models < −2.0; and the average quality of 200 highly refined X-ray structures −0.5 [±0.4]; and the model assessed using the MetaMQAPII server^42^ [Global model accuracy: GDT_TS: 81.07; RMSD: 2.2 Å]). The empirical forcefield FoldX^43^, ^44^ under the YASARA^45^, ^46^ molecular visualization program was used to estimate the free energy difference (ΔΔG) stability change upon mutagenesis from wild-type (p.Gly23Val) in silico. The FoldX “RepairPDB” option followed by “Mutate residue” was used to calculate the stability change (number of runs: 3; pH: 7; temperature: 298 K; ionic strength: 0.05 M; VdW design: 2). The resulting mean energy is expressed in kcal/mol, and the prediction decision on whether the mutation destabilizes structure is based upon Schymkowitz et al.^43^ and Guirois et al.^44^ where severely reduced structural stability ΔΔG is considered to be >1.6 kcal/mol. Intra-protein residue interactions were determined using the Protein Interactions Calculator (PIC).^47^ PyMol was used for 3D visualization, analysis, and figure preparation.

### MEF Culture

Mouse embryonic fibroblasts (MEFs) were maintained, transfected, and processed for immunofluorescence with antibodies detailed in Tables S7 and S8 as previously published.^30^ To assess endocytic trafficking, cells were transduced with 30 particles per cell of Bacman 2.0 Rab5GFP and Rab7RFP (CellLight, Molecular Probes), incubated for 18 hr in media without serum, then 100 ng/mL EGF-Alexa-647 (Molecular Probes) was added in media plus 10% serum for 10 or 15 min. Cells were fixed in 4% PFA, costained with DAPI, and mounted. Alternately, cells were transfected with Rab5^Q79L^-RFP, EGFR-GFP, or DOP-Flag,^48^ incubated in media without serum for 18 hr, then treated with ligands (100 ng/mL EGF, 5 μM DPDPE [Abcam]) for 90 min. Cells were fixed and costained with anti-EEA1 and anti-FLAG antibodies (Table S7), DAPI, and mounted.

For analysis of UPS and autophagy, cells were treated with 200 μM MG132 (Sigma) and/or 100 nM Bafilomycin-A1 (Sigma), and/or starved in EBSS media (Invitrogen) for 3 hr. Cells were rinsed twice in PBS and harvested by lysing in hot (100°C) SDS buffer (0.01 M Tris-EDTA [pH 7.5], 1% SDS). Lysates were then boiled at 100°C for 5 min, followed by brief sonication. After SDS-PAGE on a variety of NuPAGE precast gels, proteins were transferred to Nitrocellulose (Thermo Fischer Scientific) or Hybond-P membranes (GE Healthcare Life Sciences). Membranes were probed with antibodies detailed in Table S7. Images were captured using an ImageQuant LAS 4000 (GE Healthcare Life Sciences) and semiquantitative protein detection was done by ImageJ.

### UPS Activity Assay

Cerebella or MEFs were lysed in UPS buffer (10 mM Tris, 1 mM EDTA, 1 mM EGTA, 250 mM sucrose, 1.5 mM MgCl_2_, 0.05% NP40, 5 mM DTT, and 2 mM ATP). Proteasome activity was determined by incubating equal amounts of protein with 1 μM fluorescent proteasome substrates N-succinyl-Leu-Leu-Val-Tyr-7-amino-4-methylcoumarin (Suc-LLVY-AMC: Sigma), Boc-Leu-Ser-Thy-Arg-AMC (Boc-LSTR-7-AMC: Sigma), or Z-Leu-Leu-Glu-AMC (Z-LLE-AMC: Sigma) as substrates for chymotrypsin, trypsin, and caspase-like activities of the proteasome, respectively, with or without 1 nM MG132 (Sigma) for 30 min at 37°C. Data reflect kinetics of the linear phases of the curves of florigenic substrate production measured on a Perkin Elmer Victor2 multiwell plate reader.

### UPS Flux

*Ub*^*G76V*^*-GFP* (a gift from Nico Dantuma, Addgene plasmid #11941) was transfected into MEFs and mean GFP fluorescence intensity was determined by FACS 72 hr after transfection. This reporter contains the UFD signal of an N-terminal uncleavable ubiquitin moiety Ub^G76V^ that serves as target for polyubiquitylation and degradation by the proteasome.^49^

### Ubiquitin IPs

Brains were lysed in 50 mM Tris-HCl (pH 7.5), 1 mM EDTA, 1 mM EGTA, 1% (v/v) Triton-X, 0.27 M Sucrose, HALT Protease inhibitor (ThermoFisher), 1 mM DTT, 1 mM PMSF, 100 mM NEM. Halo-tagged Ubqln1, Fam63, or Eps15 UBDs were immobilized on Halo-Link Resin (Promega) overnight at 4°C, washed. then incubated with brain lysates for 2 hr at 4°C, to immunoprecipitate proteins modified with specific ubiquitin chains. Resin was washed three times in lysis buffer, boiled in 1× NuPAGE LDS Sample Buffer and Sample Reducing Agent (Thermo Fischer Scientific), and analyzed by western blot.

### Imaging and Image Analysis

Confocal images were captured with a Nikon A1R confocal microscope. Color brightfield images were captured with an Olympus Dotslide. Macroscopic images were captured on a Nikon AZ100 macroscope with a Qimaging Micropublisher 5 cooled color camera (Qimaging). Image capture was performed using in-house scripts written for IVision (BioVision Technologies). Image analysis was performed with ImageJ. For analysis of EGF in early and late endosomes, each channel was background corrected with “RollingBall” and segmented to generate a binary image. Puncta were counted using “Find Maxima.” Images were combined to display only colocalization and the number of colocalizing puncta counted using “Find Maxima.” Plotted in the figure: “early endosome” localization represents EGF colocalizing with Rab5 only, “late endosome” localization represents EGF colocalizing with Rab7 only or Rab5 and Rab7. Analysis of membrane versus luminal localization in Rab5^Q79L^ endosomes used in-house scripts (available upon request) in which the user defines the membrane and lumen based only on the red signal.

### Statistics

Statistical analyses were carried out in Microsoft Excel or GraphPad Prism6. Analysis of microarray data was performed in Affymetrix Transcriptome Analysis Console v3.0 and proteomic data in Perseus software.

## Results

### Homozygous Mutation in *PLAA* Causes a Severe Neurodevelopmental Disorder

**Figure 1:**
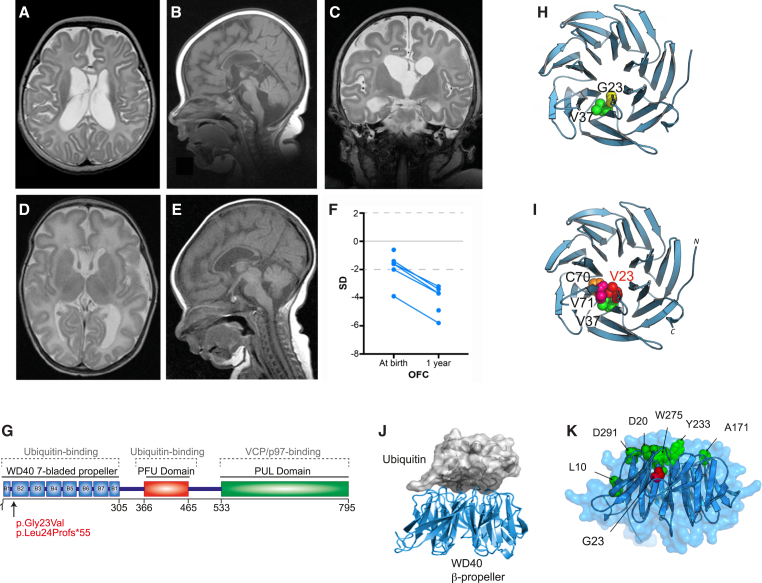
Homozygous Mutations in *PLAA* Causes a Severe, Infantile Neurodysfunction Disorder (A–E) MRI images of individuals A-IV-8 (A–C, aged 3 months) and A-IV-1 (D, E, aged 3 weeks). Axial (A, D), sagittal (B, E), coronal (C), T1-weighted (B, D, E), T2-weighted (A, C). Widespread T2-hyperintensity throughout the white matter and simplified gyral pattern frontally are evident. Ventricles and occipital horns are asymmetrically dilatated (A, C). Thinning of corpus callosum is evident (B, E). (F) Z-scores of the occipital frontal circumference (OFC) of affected individuals at birth and around a year highlight progressive microcephaly. (G) The mutations fall in the first exon, within the WD40 repeat domain of PLAA. (H) Homology model of the WD40 β-propeller domain of human PLAA, based upon the crystal structure of yeast Doa1; Gly23 is buried within the inner-most β strand 1 within blade 2 where it supports hydrophobic interaction with Val37. (I) Mutant Val23 is predicted to destabilize structure likely due to steric clashes between its side chain Cϒ1 atom with spatially proximal residues in blade 3 (labeled). (J) Crystal structure of the yeast PLAA homolog Doa1 N-terminal WD40 β-propeller (cyan) in complex with ubiquitin (white surface), adapted from Pashkova et al.^22^ (K) Location of experimentally defined key residues for ubiquitin-binding (green) with respect to p.Gly23Val (red) shown mapped on the 3D model of human PLAA WD40 β-propeller. See also Figure S1.

In three consanguineous families, seven infants presented with a severe neurodevelopmental disorder—originally diagnosed as either PEHO (progressive encephalopathy with edema, hypsarrhythmia, and optic atrophy [MIM: 260565]) or acrocallosal-like syndrome (MIM: 200990)—and were independently found to carry an identical c.68G>T (p.Gly23Val) missense substitution in *PLAA* (*Phospholipase A2-activating protein* [GenBank: NM_001031689.2]) (Figures 1 and S1), encoding a highly conserved ubiquitin binding protein. We subsequently identified an individual from a fourth consanguineous family with a homozygous c.68dupG (p.Leu24Profs^∗^55) frameshift mutation who also presented with a similar but more severe neurodevelopmental disorder.

PLAA-associated neurodevelopmental disorder (PLAAND) is characterized at birth by truncal hypotonia, increased limb tone and feeding difficulties, mildly dysmorphic facial features, and hirsutism. Progressive limb spasticity, microcephaly, and optic atrophy developed in the first year (Figure 1F). Most had seizures that began between the first week of life and 2 years (Table 1 and S1). Where electroencephalogram (EEG) data are available, electroclinical seizures with hyper-rhythmic discharges were observed. Affected children die of apnea and recurrent pneumonia by 6 years of age (range 12 days to 6 years) (Table 1 and S1). MRI brain findings in the first year included a thin corpus callosum, delayed myelination, a simple immature overall gyral pattern, particularly frontally, and large cavum septum pellucidum/vergae (Figures 1A–1E). Scans after 1 year showed features of cerebellar and cerebral atrophy.

Autozygosity mapping and exome sequencing identified the homozygous mutations within *PLAA*, which segregated in an autosomal-recessive manner in all four families (combined LOD score of 4.52 for families A–C, Figure S1). Although families A–C are not knowingly related, we inferred that the c.68G>T (p.Gly23Val) mutation was derived from a distant common ancestor, as it was inherited on the same haplotype (Table S2). The absence of either homozygous variant in ∼3,000 ethnically matched control individuals and ExAC databases indicated these were probably the pathogenic mutations. Homology modeling of the N-terminal WD40 seven-bladed β-propeller in PLAA established the p.Gly23Val variant is buried within the innermost β strand of blade 2, where it is predicted to destabilize structure (mean ΔΔG = 2.6 kcal/mol). This WD40 β-propeller domain is one of two UBDs found in PLAA, and the mutation lies close to the interface involved in high-affinity ubiquitin binding (monoUb Kd ∼220 μM)^21^, ^22^ (Figures 1G–1K).

### PLAA Is Essential for Mammalian Development and Endolysosomal Trafficking

As the function of mammalian PLAA is poorly understood, we generated a *Plaa*-null mouse model (Figures S2A and S2B). Expression studies confirmed ubiquitous expression of *Plaa*, with endogenous PLAA localizing throughout the cell, within the cytoplasm and nucleus (Figures S2C and S2D). *Plaa*^*−/−*^ embryos die in mid-gestation (Table S3), with the few mutants surviving to E15.5 being runted and anemic (Figure S2E).

**Figure 2:**
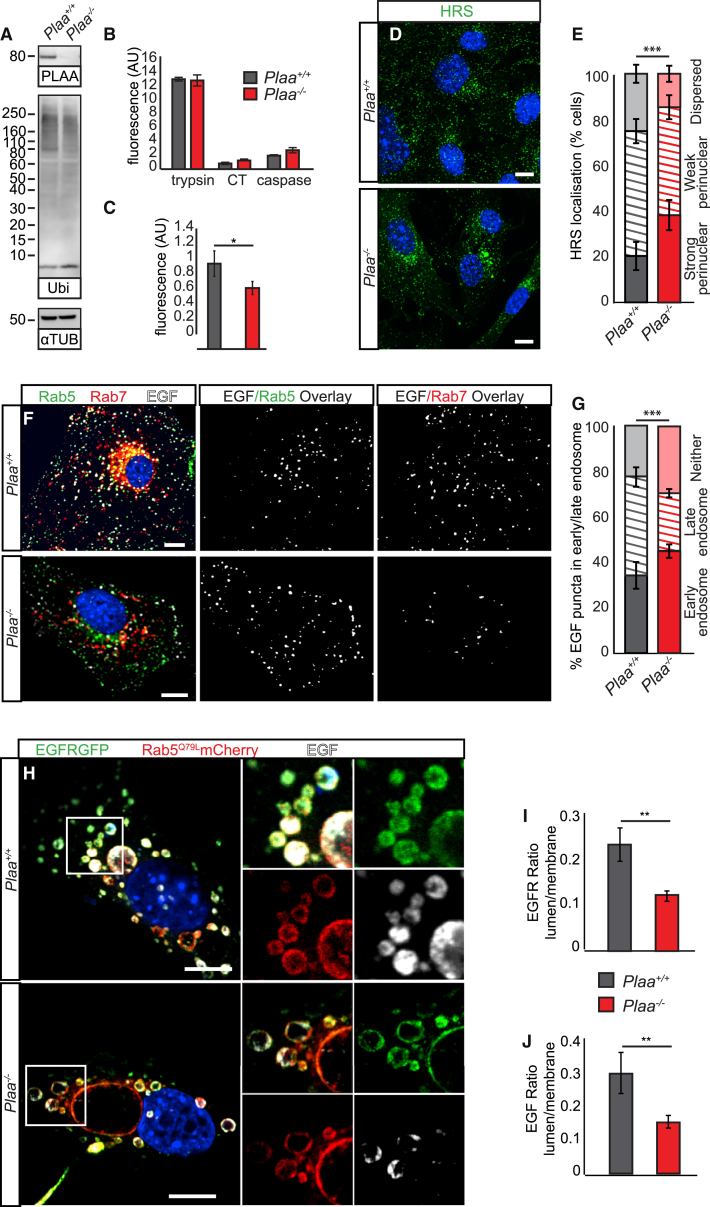
PLAA Is Required for Trafficking of Integral Membrane Receptors to Late Endosomes in an ESCRT-Dependent Manner (A) No global changes in polyubiquitin or free ubiquitin levels are detected in *Plaa*^*−/−*^ MEFs. (B) No impairment in tryptic, chemotryptic (CT), or caspase activity in *Plaa*^*−/−*^ MEFs was observed, suggesting that proteasomal activity is not compromised. (C) FACS analysis revealed reduced levels of the UFD reporter *Ub*^*G76V*^*-GFP* in *Plaa*^*−/−*^ MEFs, indicating increased UPS flux. (D and E) HRS (ESCRT-0) is mislocalized to perinuclear accumulations in *Plaa*^*−/−*^ MEFs. (F and G) In *Plaa*^*−/−*^ MEFs, EGF is internalized and reaches early endosomes (Rab5-GFP) normally, but trafficking to the late endosome (Rab7-RFP) is impaired. Colocalization of EGF and endosome markers are highlighted in white in the center and right images. (G) Quantification of EGF puncta which colocalize with Rab5-GFP (early endosome), Rab7-RFP (late endosome), or do not localize to either (neither). (H–J) *Plaa*^*−/−*^ MEFs fail to internalize EGF and its receptor (EGFR-GFP) into the lumen of Rab5^Q79L^-positive enlarged endocytic structures. (I and J) Quantification of the ratio of receptor or ligand intensity on the membrane versus lumen of the Rab5^Q79L^ endosomes. ^∗^p < 0.05, ^∗∗^p < 0.01, ^∗∗∗^p < 0.001; error bars represent SEM. n = 3 WT and n = 3 *Plaa*^*−/−*^ MEF lines in (B), (C), (E), and (G); n > 200 endosomes from 3 MEF lines per genotype in (I) and (J). Student’s t test in (C), (I), and (J); Chi squared test in (E) and (G). Scale bars represent 10 μm. See also Figures S2 and S3.

Pinpointing a functional role for mammalian PLAA is complicated by the diversity of roles described for yeast Ufd3/Doa1, including regulating free ubiquitin levels and trafficking of ubiquitylated proteins to various degradative pathways. In the absence of PLAA, no compensatory transcriptional changes in ubiquitin expression, global changes in free ubiquitin, or accumulation of polyUb chains were detected (Figure 2A). Moreover, proteasomal activity was not globally perturbed in *Plaa* mutants; in fact, decreased levels of ubiquitin-fusion degradation (UFD) proteasomal reporter Ub^G76V^-GFP suggest increased proteasomal flux in the absence of PLAA (Figures 2B and 2C). In *Plaa*^*−/−*^ mutants, we detected no compensatory upregulation of genes involved in Ub-based degradation pathways including ERAD or MAD (Figure S3A).

Integral membrane proteins undergo endocytosis and trafficking to the early endosome, where Ub-dependent sorting either recycles these proteins back to the plasma membrane or targets them for lysosomal degradation. This involves recognition of Ub-modified cargos for Endosomal Sorting Complexes Required for Transport (ESCRT)-dependent internalization into late endosomal compartments, or multivesicular bodies (MVBs), ahead of fusion with the lysosome.^50^ Ufd3 was shown to direct ubiquitylated cargo for degradation via the MVB in yeast, by directly binding ESCRT-0 subunits Hse1/STAM1 and Vps27/HRS.^27^ In *Plaa*-null cells, localization of HRS is perturbed, without disruption of endosomal or lysosomal morphology, suggesting that ESCRT-0 function may be disrupted (Figures 2D, 2E, and S3B–S3E). To test the functional integrity of ESCRT-dependent trafficking to the MVB/late endosome in the absence of PLAA, we followed the internalization of epidermal growth factor (EGF) after binding to the EGF Receptor (EGFR), which triggers endocytosis of both receptor and ligand and subsequent ESCRT-dependent trafficking to the lysosome for degradation. While initial endocytosis of EGF to the early endosome is unaffected in *Plaa*-null cells, it fails to efficiently reach the late endosome, indicating disrupted trafficking from early to late endosomes (Figures 2F and 2G). To allow visualization of distinct endosomal membrane and lumen compartments, we expressed a constitutively active Rab5^Q79L^ and followed ligand-induced internalization of EGFR and the neural G protein-coupled receptor (GPCR) δ-Opioid receptor (DOP) into the lumen of the resulting enlarged endosomes.^48^ In the absence of PLAA, these receptors/ligands remain trapped at the membrane (Figures 2H–2J, S3F, and S3G). We conclude that loss of mammalian PLAA disrupts ESCRT-0 localization and ubiquitin-dependent internalization of receptors and their ligands into MVB/late endosomes for lysosomal degradation.

### Reduced Levels of PLAA Result in Early-Onset Neural Dysfunction and Premature Lethality

**Figure 3:**
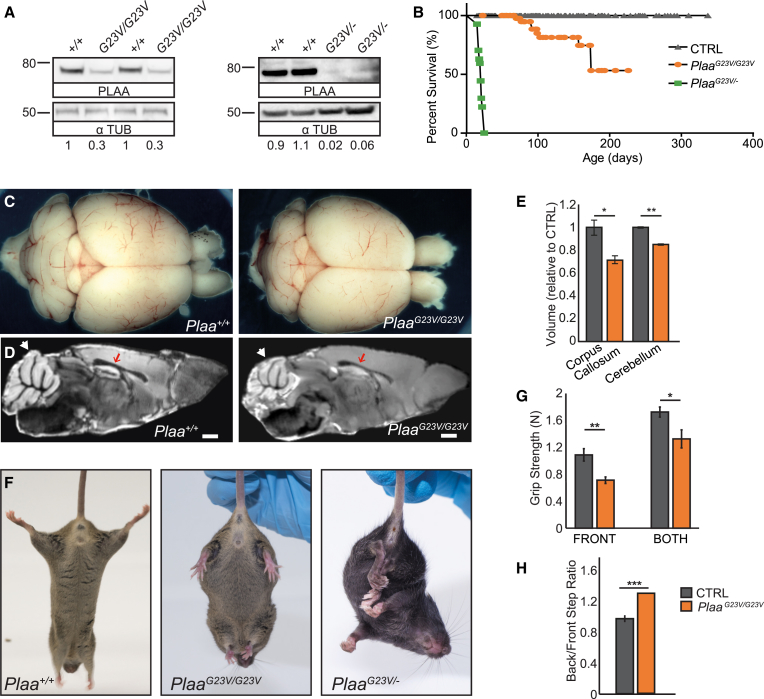
Reduction of PLAA Levels in Mouse Results in Microcephaly and Early-Onset Neural Dysfunction, Including Early Lethality, Ataxia, and Muscle Weakness (A) PLAA protein levels in the cerebellum are reduced to <30% in *Plaa*^*G23V/G23V*^ mutants and <6% in *Plaa*^*G23V/−*^ mutants (densitometry below). (B) Kaplan-Meier survival curve showing 50% of *Plaa*^*G23V/G23V*^ mice have to be culled by 6 months due to severe hindlimb paralysis or balance perturbations. *Plaa*^*G23V/−*^ mice die around weaning due to respiratory distress and paralysis. (C) Gross brain morphology of *Plaa*^*G23V/G23V*^ and *Plaa*^*+/+*^ mice at 3 months. (D and E) Representative sagittal section of MRI from 3-month-old *Plaa*^*G23V/G23V*^ and *Plaa*^*+/+*^ brains, showing reduced cerebellar (white arrow) and corpus callosum (red arrow) volume relative to total brain volume in mutants (D) quantified in (E); n = 3 CTRL (*Plaa*^*+/+*^ or *Plaa*^*G23V/+*^), n = 3 *Plaa*^*G23V/G23V*^. (F) *Plaa*^*G23V/G23V*^ and *Plaa*^*G23V/−*^ mice show neurodysfunction in the hindlimb clasp test. Whereas wild-type mice splay their hindlimbs, *Plaa*^*G23V/−*^ mice show a severe hindlimb clasping phenotype and *Plaa*^*G23V/G23V*^ mice display a partial phenotype. (G and H) *Plaa*^*G23V/G23V*^ mice show significantly reduced grip strength and significantly altered gait, resulting in an increase in the ratio of back/front step length, n = 7 CTRL (*Plaa*^*+/+*^ or *Plaa*^*G23V/+*^) and n = 5 *Plaa*^*G23V/G23V*^. Scale bars represent 1 mm. Error bars represent SEM, ^∗^p < 0.05, ^∗∗^p < 0.01, ^∗∗∗^p < 0.0001, Student’s t test. See also Figure S4.

To confirm pathogenicity of the c.68G>T human mutation and to provide an informative model of the human disease, this mutation was introduced into the orthologous mouse gene using CRISPR/Cas9 gene-editing (protein ID: 94%, Figures S4A and S4B). *Plaa*^*G23V/G23V*^ mice have a 70% reduction in PLAA protein levels by western blot (Figure 3A), confirming that the p.Gly23Val variant destabilizes protein structure. In contrast to the embryonic lethality of *Plaa*^*−/−*^ mice, *Plaa*^*G23V/G23V*^ are born at Mendelian ratios indicating this is a viable hypomorphic allele (Table S4). *Plaa*^*G23V/G23V*^ mutants exhibit early-onset neurodysfunction phenotypes, which progressively deteriorate such that 50% of mutants must be culled by 6 months (Figure 3B). Levels of PLAA abundance and/or function are further reduced in *Plaa*^*G23V/−*^ compound heterozygote mice, resulting in accelerated decline with pronounced paralysis and respiratory distress (Figures 3A, 3B, and S4C) and the pups die before weaning (P17–P21 [postnatal day 17–21]; Table S5). *Plaa*^*G23V/G23V*^ brains are smaller than littermates (Figure 3C) and MRI analysis reveals significant reductions in corpus callosum and cerebellar volumes in *Plaa*^*G23V/G23V*^ brains, similar to the features reported in imaging of the human affected individuals (Figures 1A–1F and 3D and 3E).

Tremor and motor disorders are detectable from P14 in *Plaa*^*G23V/G23V*^ mutants and P7 in *Plaa*^*G23V/−*^ compound mutants, including a range of neuromuscular weakness and hypomotility phenotypes. *Plaa*^*G23V/G23V*^ animals fail to splay hindlimbs when suspended, whereas *Plaa*^*G23V/−*^ mice displaying a more pronounced clasping phenotype (Figure 3F). *Plaa*^*G23V/G23V*^ animals also exhibit reduced grip strength, pronounced kyphosis, and muscle wasting (Figures 3G and S4D).

### PLAA Deficiency Disrupts Purkinje Cell Migration, Dendrite Arborization, and Neurotransmission

**Figure 4:**
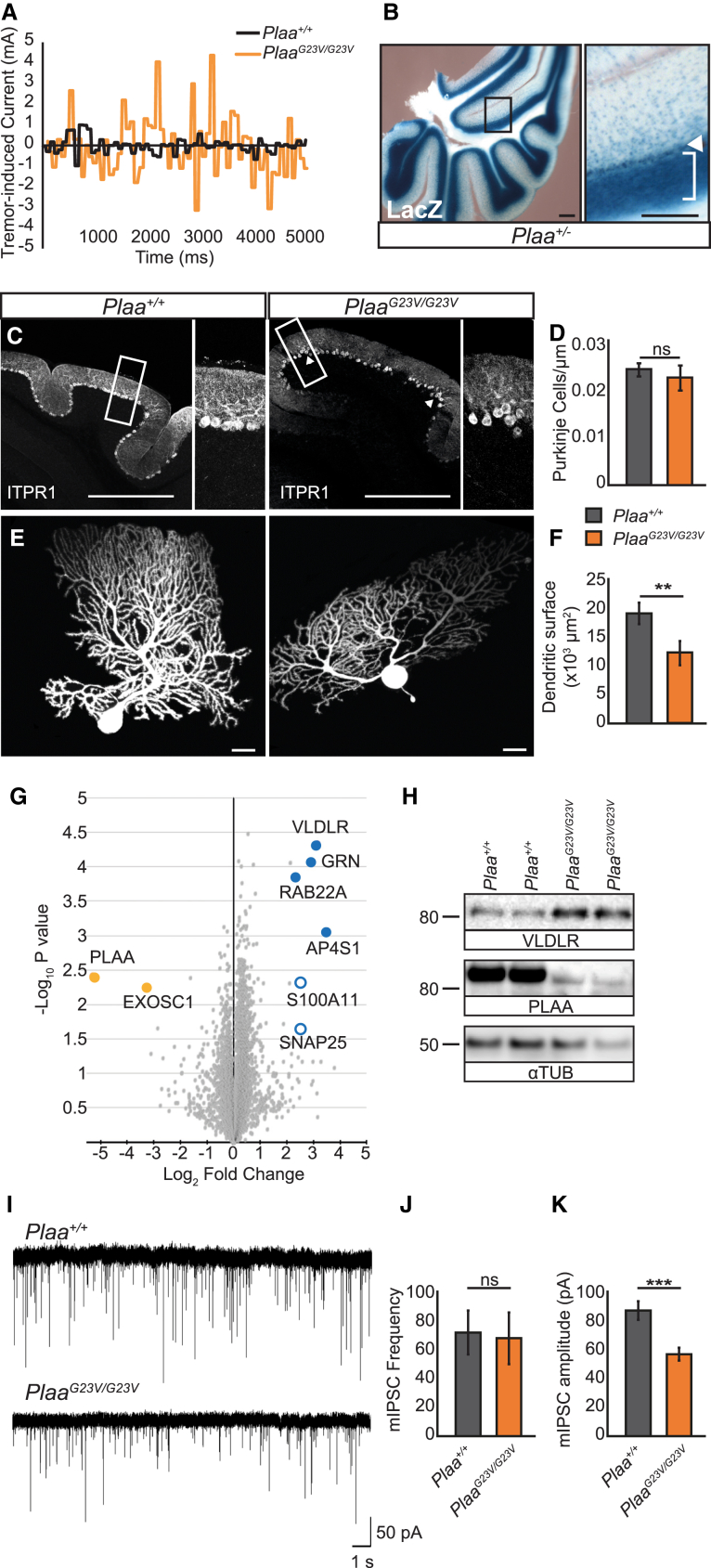
PLAA Reduction Results in Disrupted Purkinje Cell Migration and Central Synaptic Dysfunction (A) *Plaa*^*G23V/G23V*^ mice display early-onset kinetic tremor with postural aspect, detectable from before P21. (B) X-gal staining of *Plaa*^*+/−*^ brains reveal *Plaa* is highly expressed in the cerebellum, in the granular cell (bracket) and Purkinje cell (PC) (arrow) layers. (C and D) PCs (marked by anti-ITPR1) are disorganized in *Plaa*^*G23V/G23V*^ cerebella indicating disrupted PC migration although total PC density remains unchanged. (E and F) Dye filling of PCs reveals *Plaa*^*G23V/G23V*^ PCs show reduced dendritic branching, resulting in reduced dendritic surface (quantified in F) (n = 8 cells from 4 WT mice, n = 7 cells from 4 *Plaa*^*G23V/G23V*^ mice). (G) Summary of total proteome mass spectrometry analysis of *Plaa*^*+/+*^ and *Plaa*^*G23V/G23V*^ cerebella with the most statistically significant differentially expressed proteins highlighted with larger circles (blue = upregulated in *Plaa*^*G23V/G23V*^, orange = downregulated, filled circle: FDR < 0.05 t test significant, open circle: Student’s t test significant). (H) Immunoblot confirmation of VLDLR upregulation in *Plaa*^*G23V/G23V*^ cerebella. (I–K) Patch clamp recordings from PCs reveal normal frequency but reduced amplitude of mIPSCs in *Plaa*^*G23V/G23V*^ mice (quantified in J and K); n = 13 cells from 3 mice for each genotype. Scale bars represent 500 μm in (B) and (C) or 20 μm in (D). Error bars represent SEM; ns, not significant, ^∗∗^p < 0.01, ^∗∗∗^p < 0.001, Student’s t test. See also Figure S5.

Both *Plaa*^*G23V/G23V*^ and *Plaa*^*G23V/−*^ mice display altered gait, disrupted balance, and early-onset postural tremor with kinetic aspect, suggestive of central disturbances in the cerebellar motor circuits relaying information related to muscle coordination and balance (Figures 3H, 4A, and S5D; Movies S1 and S2). This type of tremor has been previously linked to early central synaptic dysfunction in rodents.^51^, ^52^ *Plaa* is expressed in the postnatal brain, with highest levels in the CA hippocampal neurons, cerebellar granular cell layer, and Purkinje cells (PCs) (Figure 4B). *Plaa*^*G23V/G23V*^ animals display a significant reduction in cerebellar volume that is more pronounced in *Plaa*^*G23V/−*^ mice, which show additional cerebellar foliation defects (Figures 3E and S5A–S5C). Transcriptome analysis of *Plaa*^*G23V/G23V*^ cerebella revealed decreased expression of PC markers, with parallel increased glial and complement-microglial markers suggesting reactive gliosis (Figures S5E and S5F). Histological and immunofluorescent analysis revealed no reduction in PC density in *Plaa*^*G23V/G23V*^ cerebella (Figures 4C and 4D); instead, PCs fail to form a uniform layer in *Plaa*^*G23V/G23V*^ cerebella, consistent with a defect in migration. *Plaa*^*G23V/G23V*^ PCs also show abnormal dendritic branching (Figures 4E and 4F). Together these results support a role for PLAA during PC development, as opposed to degeneration.

To address how disrupted sorting of Ub-modified cargos may underlie the mutant cerebellar phenotype, we undertook an unbiased whole-proteome analysis of early symptomatic *Plaa*^*G23V/G23V*^ cerebella (aged 3 months). We found no changes in protein levels suggestive of deregulated MAD (i.e., mitochondrial function or turnover) or UPS (i.e., proteasomal subunits) degradation in the absence of PLAA; nor did we find evidence of cell loss (apoptosis). In contrast, all proteins significantly increased in mutant cerebella (>5-fold, FDR < 0.05) were involved in vesicular trafficking (AP4S1, SNAP25, RAB22A, S100A11) or were receptors/ligands trafficked via the endolysosomal pathway (VLDLR, GRN)^53^, ^54^, ^55^, ^56^, ^57^, ^58^ (Figures 4G and 4H). Immunoblot confirmed the upregulation of VLDLR, SNAP25, and AP4S1 (Figures 4H and S5H–S5J), while transcriptional analysis confirmed this upregulation was post-transcriptional (Figure S5G). VLDLR (Very low-density lipid receptor) is required for cerebellum development and PC migration.^59^ The defects in PC migration and dendrite maturation, as well as the ataxia and tremor present in *Plaa*^*G23V/G23V*^ mice may, therefore, result from the accumulation of dysfunctional VLDLR due to disrupted post-endocytic trafficking to the lysosome.

To assess the functional competence of the PCs in *Plaa*^*G23V/G23V*^ cerebella, we performed whole-cell patch-clamp recordings and analyzed spontaneous miniature inhibitory post-synaptic currents (mIPSCs). Reduced amplitude of mIPSCs, with no effect on frequency or decay kinetics, was observed in *Plaa*^*G23V/G23V*^ PCs, consistent with a functional deficit in cerebellar outputs, which may underlie the tremor and cerebellar ataxia observed in these mice (Figures 4I–4K).

### PLAA Is Required for Efficient Synaptic Vesicle Recycling at NMJs

**Figure 5:**
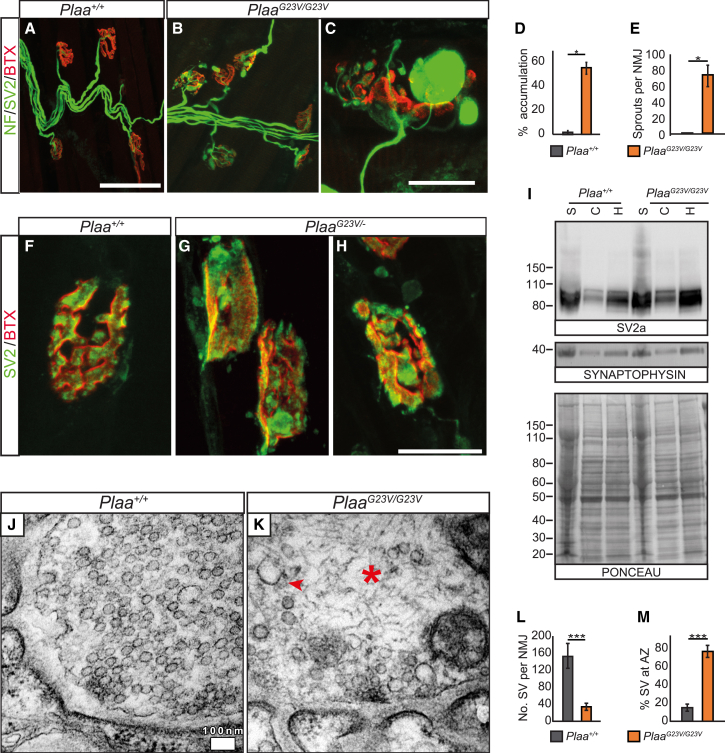
PLAA Reduction Results in Disrupted SV2 Degradation and Reduced Synaptic Vesicles (A–E) NMJs on LAL muscles in 3-month-old *Plaa*^*G23V/G23V*^ mice show striking pre-synaptic swellings (quantified in D) or sprouting (quantified in E). Mann-Whitney U test, n = 3 mice per genotype; scale bar represents 60 μm in (A) and (B), 18 μm in (C). Error bars represent SEM. (F–H) *Plaa*^*G23V/−*^ NMJ at P15 show abnormal accumulations of SV2 (green) compared to controls. Scale bar represents 18 μm. (I) Synaptic preps from cerebella show SV2 levels are increased in *Plaa*^*G23V/G23V*^ mutants (S, synaptic; C, cytoplasmic; H, homogenate). (J–M) Transmission electron microscopy of synaptic boutons on LAL muscles from 3-month-old *Plaa*^*+/+*^ (J) and *Plaa*^*G23V/G23V*^ (K) mice, quantified in (L) and (M). Scale bar represents 100 nm. Mutant synaptic boutons have reduced SV numbers (L), with the reduction more pronounced in the reserve pool and remaining SVs limited to periphery close to active zones (AZ) (M). *Plaa*^*G23V/G23V*^ synapses show structured neurofilament accumulations (asterisk) and prevalent abnormal large endosomal structures are evident (arrowhead). Abbreviations are as follows: NF, neurofilament; SV2, Synaptic vesicle 2; BTX, Bungarotoxin. ^∗^p < 0.05, ^∗∗^p < 0.01, ^∗∗∗^p < 0.001. See also Figure S6.

In addition to central defects, the muscle weakness and wasting observed in *Plaa*^*G23V/G23V*^ mice may reflect involvement of the peripheral nervous system. Analysis of *Plaa*^*G23V/G23V*^ neuromuscular junctions (NMJs) revealed that every endplate was fully innervated. However, there were increased numbers of endplates with terminal swellings and/or sproutings, typically a compensatory response to a poorly functioning synaptic terminal^60^ (Figures 5A–5E and S6A–S6G). *Plaa*^*G23V/G23V*^ muscle fibers had decreased diameter, consistent with atrophy (Figure S7A). NMJ disruption is detected as early as P14 in *Plaa*^*G23V/−*^ mice (Figures S6D–S6G). While the bulk of the presynaptic swelling is accumulation of neurofilament (NF), increased intensity and number of foci of synaptic vesicle protein 2 (SV2) were clear in *Plaa*^*G23V/−*^ NMJs suggesting a disruption in distribution and/or composition of synaptic vesicles (SV) (Figures 5F–5H and S6D–S6G). Immunoblotting confirmed synaptic accumulation of SV2 with an increased high molecular weight smear in *Plaa* mutants, consistent with disrupted SV2 degradation (Figure 5I). Furthermore, PLAA itself was detected in synaptic preparations, supporting a putative direct function in regulating Ub sorting at the synapse (Figures S5I and S5K).

Indeed, transmission electron microscopy (TEM) of *Plaa*^*G23V/G23V*^ levator auris longus (LAL) NMJs revealed profound decreases in SV numbers with increased enlarged endosomal and vacuolar structures (Figures 5J–5L and S6H). Several pools of SVs exist, including the reserve, recycling, and readily releasable pools, with distinct functional properties and modes of regeneration, some involving endocytic intermediates.^4^ A greater reduction in SVs not tethered at the active zone (perhaps representing the recycling or reserve SVs) was evident in *Plaa*^*G23V/G23V*^ NMJs (Figure 5M), which, together with the presence of prominent enlarged endosomal and vacuolar structures, suggests that Ub-mediated sorting via PLAA is required for efficient SV biosynthesis or recycling.

**Figure 6:**
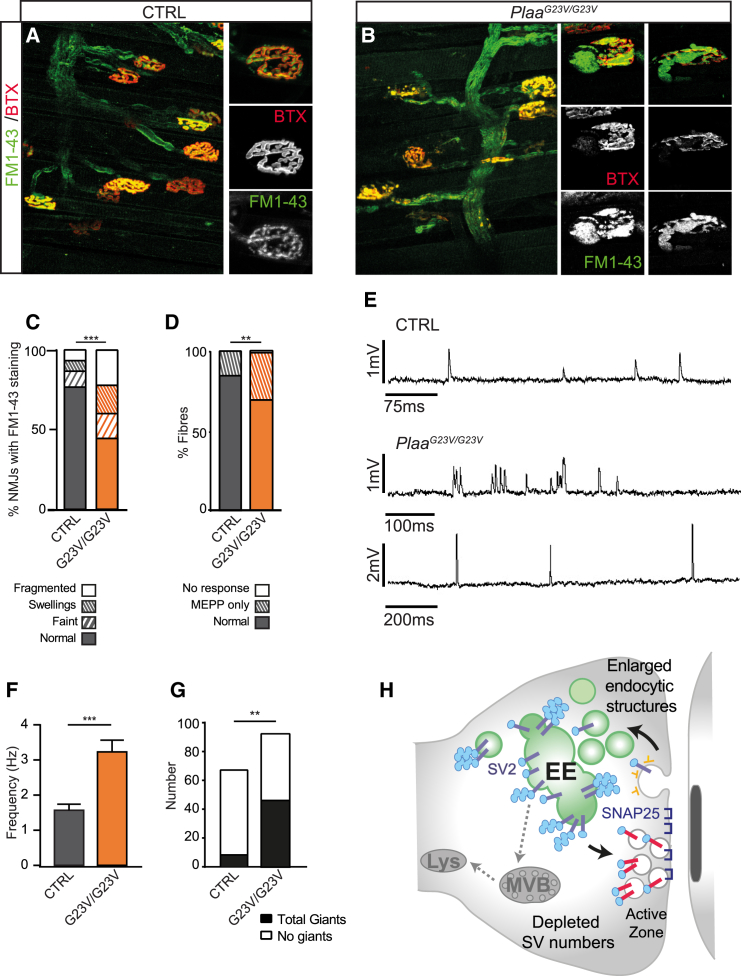
SV Trafficking and Neurotransmission Is Disrupted in *Plaa* Mutant NMJs (A–C) Motor nerve terminals in LAL muscles from 7-month-old mice were vitally stained with FM1-43. Defects are quantified in (C), n = 2 control (*Plaa*^*+/G23V*^) and n = 4 *Plaa*^*G23V/G23V*^ Chi squared test. (D) Nerve-evoked endplate potentials (EPPs) recordings showed that roughly 40% of LAL mutant fibers failed to respond. Filled bar, response to stimulation; hatched bar, no response to stimulation but spontaneous miniature endplate potentials (MEPPs) present; unfilled bar, no response to stimulation or MEPPs. n = 2 control (*Plaa*^*G23V/+*^) and n = 4 *Plaa*^*G23V/G23V*^, Fisher’s exact test. (E) MEPPs occurred in many *Plaa*^*G23V/G23V*^ NMJs at abnormally high frequency. (F) Student’s t test. (G) The incidence of spontaneous MEPPs with amplitudes more than twice the mean (“giant” MEPPs) was also significantly higher in *Plaa*^*G23V/G23V*^ NMJs from LAL. n = 2 control (*Plaa*^*G23V/+*^) and n = 4 *Plaa*^*G23V/G23V*^ (10–30 fibers sampled per muscle), Chi squared. (H) Summary model depicting how disruption of ubiquitin signaling impairs endolysosomal trafficking of synaptic membrane proteins in *Plaa* mutant neurons, leading to reduced synaptic vesicle numbers and altered neurotransmission. See also Figure S7.

To scrutinize SV recycling directly, we stained motor nerve terminals in LAL muscles with FM1-43, which selectively labels recycling SVs and other endocytic compartments in an activation-dependent manner. The general innervation pattern appeared normal, suggesting that most terminals recycled vesicles sufficiently to sustain neuromuscular transmission (Figures 6A and 6B). However, about 60% of motor nerve terminals in *Plaa*^*G23V/G23V*^ animals displayed abnormal FM1-43 uptake. This included localized swelling of terminal boutons or punctate/fragmented intense staining, consistent with the presence of enlarged endocytic structures seen by TEM (Figures 6A–6C and 5J and 5K) and suggesting defective coupling of SV fusion and recycling.

Intracellular recordings further revealed that upon nerve stimulation a significant number of *Plaa*^*G23V/G23V*^ NMJs failed to respond or gave intermittent response (Figure 6D). Of the *Plaa*^*G23V/G23V*^ NMJs which responded, EPP (end-plate potential) characteristics appeared normal (Figures S7F–S7I). The mean frequency of spontaneous MEPPs (miniature end-plate potentials) was increased in *Plaa*^*G23V/G23V*^ NMJs, together with an increased half-decay time in some muscle fibers, indicating altered synaptic function in *Plaa*^*G23V/G23V*^ NMJs (Figures 6E, 6F, and S7C–S6E). The incidence of spontaneous MEPPs with amplitudes more than twice the mean (“giant” GMEPPs) was also significantly higher in *Plaa*^*G23V/G23V*^ muscles (Figure 6G). In conclusion, our results suggest that the infantile neurodysfunction is a result of defective SV recycling and synaptic function (Figure 6H).

### Reduction of PLAA Leads to Impaired Trafficking of K63-Ubiquitylated Substrates

**Figure 7:**
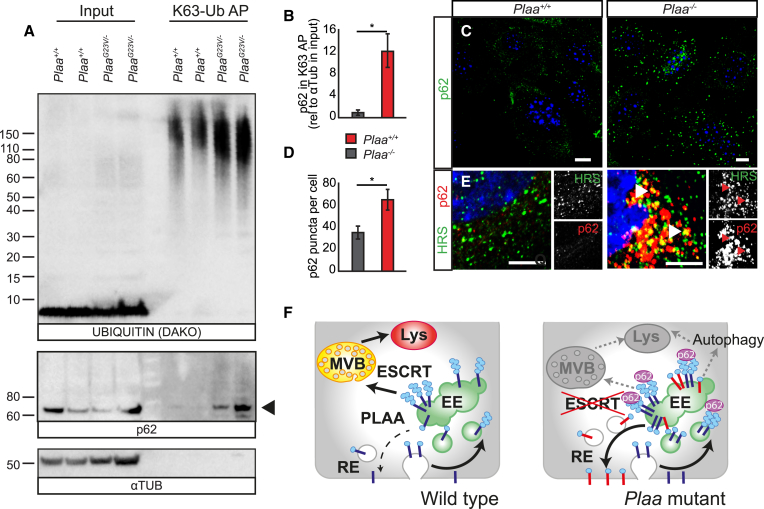
Reduction of PLAA Leads to Impaired Trafficking of K63-polyUb Substrates (A and B) Affinity purification of P17 cerebellar lysates using the K63-specific UBD (EPS15) revealed a significant accumulation of K63-ubiquitylated proteins in *Plaa*^*G23V/−*^ mutants. Blot representative of n = 3 animals per genotype. Reduction of PLAA leads to increased binding of p62 to these increased K63-ubiqutitylated substrates (quantified in B). (C–E) *Plaa*^*−/−*^ MEFs show significant increase in p62 foci (quantified in D), some of which colocalizes with mislocalized HRS (E) (see Figure S7G for zoomed out image). Scale bars represent 10 μm. Error bars represent SEM; ns: not significant, ^∗^p < 0.05, Student’s t test. (F) Schematic representation of PLAA-dependent trafficking defects through the endolysosomal system. PLAA is required for sorting of Ub-modified membrane proteins into the lumen of MVB/late endosomes. Cargos become trapped on limiting membranes of abnormal early endosome intermediates in *Plaa* mutants where they are concentrated by p62 adaptor protein for alternate lysosomal degradation via autophagy. Alternately these proteins targeted for degradation may be re-routed via the recycling endosomes to the cell surface where they may be functionally compromised (red). See also Figure S8.

To further characterize how intracellular trafficking defects could underlie the phenotypic changes in *Plaa* mutant brains, we investigated whether all polyUb species accumulate as a result of general disruption in degradation or whether specific subset of polyUb substrates are affected when PLAA function is reduced. Similar to null MEFs, no compensatory changes in UPS subunit levels or UPS flux are observed in *Plaa*^*G23V/G23V*^ cerebella, suggesting no general disruption of Ub-based degradation (Figures S8A and S8B). Using brains from wild-type and *Plaa*^*G23V/−*^ cerebella at P17, we took advantage of recently characterized small recombinant UBDs for binding pan-Ub or with high selectivity to either K63- or K48-linked polyUb.^61^, ^62^ While not readily detectable without enrichment, significant and specific accumulation of K63-polyUb species is observed in *Plaa*^*G23V/−*^ mutant brains while only minor changes in pan-Ub or K48-polyUb modified cargos are seen (Figures 7A and S8C–S8E). This is consistent with a specific primary defect in post-endocytic degradation of ubiquitylated membrane proteins, as K63-polyUb is key for internalization of receptors into the lumen of MVBs and targeting to the lysosome for degradation.^63^, ^64^, ^65^ Importantly, this is seen as early as P17, further supporting a role in normal neuronal function for K63-ubiquitylated proteins, distinct from that in age-related proteotoxic neurodegeneration resulting from compromised UPS.

As p62 binds polyubiquitylated proteins, preferentially to K63-linked species and targets them for degradation via the autophagy pathway, we tested presence of p62 in a capture of K63-polyUb.^66^, ^67^ Interestingly, we see increased binding of the autophagy adaptor p62 to these accumulated K63-linked polyUb-modified proteins in *Plaa*^*G23V/−*^ cerebella (Figures 7A and 7B). Furthermore, the number of p62 foci was increased in *Plaa*-null cells (Figures 7C and 7D), colocalizing with aberrantly localized HRS-positive endocytic structures (Figures 7E and S8G). To assay whether the accumulation of p62 foci is a result of disrupted flow through autophagy intermediates, we used the reporter GFP-RFP-LC3 in *Plaa*^*−/−*^ cells. This reporter allows the concomitant assessment of the total autophagosome pool size before and after fusion as this tandem tagged reporter labels autophagosomes (RFP^+^ and GFP^+^) as well as autophagolysosomes (RFP^+^; GFP^−^ due to pH sensitivity of GFP). *Plaa*^*−/−*^ cells show an overall increase in the pool size of both LC3-positive structures, but no change in the ratio of autophagosomes (yellow) to autophagolysosomes (red) (Figures S8F and S8H–S8J). This suggests that in the absence of PLAA, there is an increase in basal autophagy without clear defects in fusion events. Reducing PLAA function impairs endolysosomal trafficking, which could trigger p62 recruitment to accumulating K63-linked polyUb proteins on endosomes. We suggest that p62 attempts to reroute this cargo for autophagic clearance, but this rerouting via selective autophagy is much less efficient and/or compromised such that reduction in PLAA disrupts ubiquitin-dependent signaling events key for neural development and synaptic function.

## Discussion

### PLAA Is Essential for Post-Endocytic Degradation of K63-Ubiquitylated Cargo

The functional outcome of ubiquitylation is determined by how ubiquitin signals are interpreted by a large number of ubiquitin binding domain proteins, vastly increasing the potential biological applications for this post-translational modification (PTM). However, this complicates assigning specific functions to widely expressed adaptor proteins like PLAA/Ufd3/Doa1, which have multiple distinct UBDs and no catalytic activity. Indeed, the yeast ortholog Ufd3 has been implicated in protein quality control through diverse Ub-based sorting mechanisms, many of which involve interaction with p97/VCP segregase, including ERAD, UPS, and MAD.^21^, ^25^ Unlike in yeast, loss of mammalian PLAA does not affect levels of free ubiquitin, nor do we detect accumulation of high-molecular-weight polyUb species associated with impaired UPS- or mitochondrial-associated degradation, which are frequently associated with neurodegeneration. In yeast, Doa1 has been linked to ribophagy, starvation-induced degradation of the 60S ribosome.^26^ Recently mammalian PLAA, together with VCP/p97, has been implicated in stress granule assembly, mRNA-protein aggregates that form during translational disassembly induced during stress,^18^ as well as lysophagy, which involves the clearance of damaged lysosomes by autophagy.^68^ However, these studies focused on the role of PLAA in response to various cell stressors, so any homeostatic role of PLAA, for example during development, remained unclear.

In this study we demonstrate a conserved role for mammalian PLAA in Ub-mediated trafficking of membrane proteins though the endolysosomal pathway. We demonstrate that PLAA is essential for mammalian embryonic development. *Plaa*^*G23V/G23V*^ mice (homozygous for the human mutation in families A–C) survive past weaning, so we conclude that PLAAND-affected individuals possess hypomorphic *PLAA* mutations, as we would not expect null mutations to be compatible with life. The c.68G>T (p.Gly23Val) missense mutation introduces steric clashes in the WD40 ubiquitin binding domain, which destabilizes PLAA protein. Whether this additionally disrupts ubiquitin binding, directly leading to defects in ubiquitin-based trafficking, was not addressed; the significant reduction in protein levels probably accounts for the defects observed, including slowed removal of target proteins from membranes leading to the observed K63 accumulation. The c.68dupG (p.Leu24Profs^∗^55) insertion is in exon 1 of *PLAA* and so may escape nonsense-mediated decay;^69^ it is possible that translation begins at the downstream methionine (Met58), which would result in a N-terminally truncated PLAA protein, missing part of the WD40 propeller, which would likely also be highly destabilizing.

After this manuscript was submitted, Zaccai et al.^70^ published a missense c.2254C>T mutation in human *PLAA* resulting in a non-destabilizing p.Leu752Phe change in the PUL domain, causing a similar but milder clinical phenotype, which they diagnosed as leukoencephalopathy. While their emphasis among their older cohort was on white matter abnormality, the affected individuals we describe in this study, who are younger in comparison, also have evidence of white matter involvement (i.e., delayed myelination). It is conceivable that had they survived, they may have progressed to a comparable level of leukodystrophy. Using our allelic series of mouse mutants, we demonstrate a strongly dose-dependent requirement for functional PLAA in neuronal function, brain development, and viability. Together, these data support use of the unifying term PLAA-associated neurodevelopmental disorder (PLAAND), which covers the phenotypic spectrum of human neurological disease resulting from different *PLAA* mutations. While the Zaccai paper focused on misregulation of Phospholipase A2 activity and subsequent disruption of downstream Prostaglandin E_2_ induction as causative,^70^ we see no alteration in Phospholipase A2 (PLA2) activity in our most severely affected *Plaa* mutant brains (Figure S9).

We show that PLAA is required for ubiquitin-dependent trafficking of receptors from early to late endosomes/MVBs. In neurons, reducing PLAA function disrupts synaptic structure and synaptic vesicle recycling, resulting in impaired synaptic function, as demonstrated by electrophysiology and gross phenotypes (tremor, ataxia, neuromuscular weakness). We suggest that many of these phenotypes are due to disrupted ESCRT-mediated endocytic sorting. Indeed, a spontaneous destabilizing mutation in ESCRT-0 component *Hrs*, *teetering* (*Hrs*^*tn/tn*^), results in early-onset neuromuscular weakness and hypokinesis as observed in *Plaa*^*G23V/G23V*^ mice, with accumulations of ubiquitylated synaptic proteins and disrupted SV recycling.^19^

Consistent with a conserved role for PLAA in ESCRT-dependent targeting of ubiquitylated membrane receptors for lysosomal degradation, we see accumulation of K63-Ub proteins in *Plaa* mutant brains. K63-linked Ub chains are thought to be required for internalization into the MVB lumen and, accordingly, ESCRT-0 proteins show preferential binding affinity to K63-Ub chains.^63^, ^65^ Accumulation of K63-ubiquitylated cargo has been reported in late-onset neurodegenerative disorders such as Huntington disease (MIM: 143100).^71^ We detect an accumulation of a subset of K63-ubiquitylated proteins in mutant cerebella in mice as young as P17. The very early-onset neural dysfunction (evident from birth in human and at least P7 in mouse) is distinct from classical neurodegeneration, characterized by age-related aggregation or cellular inclusions of ubiquitylated proteins.^72^, ^73^ This implicates PLAA-directed K63-ubiquitin trafficking in neuronal development. The abnormal Purkinje cell (PC) migration, dendritic tree morphology, and impaired VLDLR degradation we see in *Plaa* mutant mice is consistent with disrupted neuronal development. VLDLR is known to undergo ubiquitin-dependent endolysosomal degradation in response to Reelin signaling, where it controls PC migration.^57^, ^59^ Moreover, human mutations in *VLDLR* (MIM: 192977) are found in affected individuals with cerebellar ataxia and intellectual disability^74^ (MIM: 224050). Interestingly, at the top of our unbiased total proteomic analysis of *Plaa* mutant cerebella were several proteins encoded by human neurological disease genes with phenotypes overlapping with PLAAND (4 out of the top 6: *SNAP25* [MIM: 616330], *VLDLR* [MIM: 224050], *AP4S1* [MIM: 614706], and *GRN* [MIM: 614706]), suggesting that Reelin signaling is unlikely to be the only signaling pathway disrupted upon PLAA reduction.

As well as targeting proteins for lysosomal degradation through the endosomal pathway, K63-Ub chains have been implicated in autophagy-mediated lysosomal degradation, through autophagy adaptor protein p62, which shows binding preference for K63-Ub chains.^66^, ^67^ It has recently been proposed that PLAA, together with VCP/p97, is recruited to damaged lysosomes to promote lysophagy, downstream of K63-linked polyUb and p62.^68^ Our results are consistent with the proposal that PLAA acts downstream of p62 and K63-Ub in rerouting cargo, but we do not see evidence of lysosomal damage, nor defects in autophagolysosome fusion in *Plaa* mutants. Instead, *Plaa*-null cells display a marked increase in p62 foci, colocalizing with aberrant HRS-positive endosomal structures. We propose that p62 attempts to consolidate and reroute K63-ubiquitylated cargo trapped at the endosome to the lysosome via the autophagy pathway. Increasing autophagy pharmacologically or genetically can ameliorate neurodegenerative conditions such as ALS (MIM: 105400), Huntington, and Parkinson disease (MIM: 168600),^75^, ^76^ raising the possibility that pharmacomodulation of autophagy could also be a therapeutic option for early synaptic dysfunction observed in PLAAND and related disorders.

### PLAA Regulates Sorting of Synaptic Membrane Proteins Necessary for Synaptic Function

In addition to regulating neuronal signaling during brain development, PLAA-dependent ubiquitylated cargo sorting is required for synaptic structure and function. SVs undergo repeated cycles of exocytosis, endocytosis, and vesicle reformation: they are in essence specialized cycling endosomes. In order to maintain their precise identity, specific mechanisms must enable the sorting of SV proteins during recycling to preserve their composition and target old or damaged proteins for degradation. Our studies identify ubiquitin-mediated sorting of synaptic membrane components by PLAA as an essential feature of both central and peripheral synapses.

Ubiquitin has been shown previously to play a key role in synaptic development,^77^, ^78^, ^79^ but these studies focused on the importance of regulating levels of ubiquitin locally at the synapse. Synapses are particularly vulnerable to fluctuations of ubiquitin levels as ubiquitin is synthesized at a distance in the cell body and slowly moved by axonal transport to the synapse.^80^ The levels of ubiquitin at the synapse reflect a balance of distant synthesis with local degradation by the proteasome. This degradation is monitored by proteasome-associated DUBs like USP14, which trim polyUb chains prior to degradation of conjugated substrates to maintain free ubiquitin levels. Unlike *Usp14* mutants that show disturbances in ubiquitin homeostasis at the synapse,^79^, ^81^ *Plaa* mutants show no perturbation in synaptic-free ubiquitin levels but still have pronounced disruption of activation-based endocytosis, SV numbers, and synaptic membrane protein content. We argue that these observations support a primary role for ubiquitin signaling in endosomal sorting at synapses, which is necessary for synaptic plasticity. Importantly, it further suggests that neurodysfunction need not arise from deregulated synaptic proteostasis via the ubiquitin-proteasome system, but instead through disruptions to the endolysosomal degradative route.^17^, ^82^, ^83^ Independent support for this comes from recent extensive quantitative proteomics studies demonstrating that Ub-mediated degradation of the majority of synaptic proteins is not by the proteasome, but via an alternate route.^20^

Without signs of denervation or neuron loss, clear presynaptic changes occur in *Plaa* mutant neuromuscular junctions (NMJs). A reduction in SVs, particularly those not at the active zone, which may represent the reserve and/or recycling pool, is accompanied by accumulation of enlarged endocytic structures. The abnormal trafficking and accumulations of endocytic dye FM1-43, which marks recycling SVs, demonstrates that although initial endocytosis is not dependent on PLAA, subsequent sorting during recycling is. SV component SV2 accumulates in *Plaa* mutant synapses, suggesting that its selective degradation is impaired. Functional synaptic defects observed at mutant NMJs are consistent with a presynaptic defect where docked vesicles of variable sizes compared to controls are found. Indeed, the occurrence of giant miniature endplate potentials (GMEPPs) has been suggested to represent constitutive neurotransmitter release via endosome structures at the synapse.^84^ The occurrence of GMEPPs, together with increased MEPP frequency, is also observed in synapses lacking SNAP25, which is misregulated in *Plaa* mutant synapses.^5^

There is some evidence that a ubiquitin-based sorting mechanism may operate within an endosome intermediate at synapses to regulate the molecular composition and functionality of SV. Active zone proteins Bassoon and Piccolo control SV protein turnover, including SNAP25 and SV2, by restricting local activity of the E3 ligase SIAH1. Depletion of these components in hippocampal neurons leads to increased endolysosomal structures and accelerated degradation of SV components.^85^ In *skywalker* mutant flies, excessive endosomal SV recycling and accelerated clearance of Ub-tagged SV proteins leads to increased numbers of super-functional “rejuvenated” SVs.^86^, ^87^ Too much activity results in neurodegeneration which can be rescued by mutating components of the ESCRT^87^ or VPS C/HOPS complex,^86^ indicating that enhanced targeting of SV cargos for lysosomal degradation underlies the *skywalker* phenotype. Human ortholog *TBC1D24* (MIM: 613577) is mutated in DOOR syndrome (MIM: 220500), where affected individuals have early-development seizures and neurological involvement including optic atrophy and MRI abnormalities.^88^, ^89^ This GTPase activating protein negatively regulates the SV-associated RAB35, which associates with ESCRT-0 HRS in an activity-dependent manner to stimulate endolysosomal degradation of a subset of synaptic membrane proteins, including SV2 and SNAP25.^1^, ^87^ This suggests that turnover of different SV proteins is differentially regulated and probably involves specific ubiquitin-adaptors (as well as Rabs). Our study is the first ubiquitin adaptor protein to be directly linked to this process. We propose that PLAA plays a key role in recognizing Ub-modified, likely K63-modified, SV components and targeting them for ESCRT-dependent degradation via the MVB. The precise nature and kinetics of the ubiquitin code being read on specific SV cargos during vesicle recycling remains to be addressed: is it linkage specific or do additional domains exist on modified cargos that are read by adaptors like PLAA? Intriguingly, endogenous mammalian PLAA was recently shown to bind multiple ubiquitin chains, including K63 and K48 in a cell type-dependent manner, possibly due to cell type-specific PTMs or differentially expressed adaptor proteins.^90^ PLAA was also found to bind to poorly characterized ubiquitin linkages K6, K11, and K29 whose roles in neural biology and synaptic function are currently unknown. As improved tools to study these atypical chains in vivo are developed, the complexities of the ubiquitin code in neural biology can be unpicked.

This study demonstrates a critical role for ubiquitin signaling in synaptic function by directing turnover of synaptic membrane proteins locally via the endolysosome. Critically, from a therapeutic perspective better understanding of how ubiquitin signals regulate the fate of SV cargo could lead to the development of small molecule ubiquitin signaling modulators to enhance (in PLAAND-affected individuals) or suppress (in DOOR-affected individuals) SV component turnover, rescuing synaptic function and minimizing neuronal loss.

## Figures and Tables

**Table 1:** Summary of Clinical Features in PLAAND

**Clinical Feature**	**Fraction of Affected Individuals Displaying Feature**
**Development**	

Absent gross motor	10/10
Absent fine motor	10/10
Absent social	10/10
Absent language	10/10
Cognitive impairment	10/10

**Neurological Findings**	

Generalized seizures	8/10
Central hypotonia	8/10
Peripheral hypertonia	9/10
Bulbar symptoms	7/10
Optic atrophy	3/5^a^
Nystagmus	4/9^b^
Progressive microcephaly	9/9^b^

**Physiological**	

Dorsal edema of hands/feet	4/10
Dysmorphic facies	10/10

Related to Figure 1.

a Five children died at too early an age to have developed this feature

b One child died at too early an age to have developed this feature

## Accession Numbers

Transcriptomic microarray data are available from GEO: GSE74735. Total proteomic data are available via ProteomeXchange: PXD003140.

## Web Resources

Ensembl Genome Browser, http://www.ensembl.org/index.html
ExAC Browser, http://exac.broadinstitute.org/
FungiDB, http://fungidb.org/fungidb/
GenBank, http://www.ncbi.nlm.nih.gov/genbank/
ImageJ, https://imagej.nih.gov/ij/
Mouse Genome Informatics, http://www.informatics.jax.org/
OMIM, http://www.omim.org/
ProteomeXchange, http://www.proteomexchange.org/
PyMOL, http://www.pymol.org
RCSB Protein Data Bank, http://www.rcsb.org/pdb/home/home.do

## Supplemental Data

Document S1. Supplemental Note, Figures S1–S9, and Tables S1–S5 and S7–S9

Movie S1. *Plaa*^*G23V/G23V*^ Animal Aged 7 Weeks Displaying Disrupted Balance, Ataxia, and Tremor Related to Figure 3.

Movie S2. *Plaa*^*G23V/-*^ Animal (Agouti) and Control Littermate (Black) Aged P20 *Plaa*^*G23V/-*^ animal displays disrupted balance, ataxia and tremor. Related to Figure 3.

Table S6. *Plaa*^+/+^ or *Plaa*^*G23V/G23V*^ cerebellum protein expression profiling

Document S2. Article plus Supplemental Data
